# Correlation of Albumin, Red Cell Distribution Width and Other Biochemical and Hematological Parameters with Glycated Hemoglobin in Diabetic, Prediabetic and Non-Diabetic Patients

**DOI:** 10.3390/ijms25158037

**Published:** 2024-07-23

**Authors:** Argyrios Ginoudis, Stavroula Ioannidou, Georgia Tsakiroglou, Konstantina Kazeli, Eleni Vagdatli, Evgenia Lymperaki

**Affiliations:** 1 School of Veterinary Medicine, Aristotle University of Thessaloniki, 54124 Thessaloniki, Greece; agkinou@vet.auth.gr; 2 Department of Biomedical Sciences, International Hellenic University, 57400 Thessaloniki, Greece; stayroyla.ioannidou@gmail.com (S.I.); tsakgeorgia@gmail.com (G.T.); kkazeli@physics.auth.gr (K.K.); evagdatli@gmail.com (E.V.); 3 Hippokration General Hospital of Thessaloniki, 54642 Thessaloniki, Greece

**Keywords:** albumin, diabetes, glycated hemoglobin, mean platelet volume, red blood cell distribution width, sodium, urea

## Abstract

Diabetes mellitus is a chronic metabolic disease that affects more than 10.5% of the world’s adult population. Biochemical and hematological parameters, such as albumin (ALB) and red cell distribution width (RDW), have been shown to be altered in diabetic patients. This study aimed to correlate hematological and biochemical parameters with glycated hemoglobin (HbA1c). A total of 777 adults (372 women and 405 men, aged 19–85 years) were divided into three groups: 218 participants with HbA1c < 5.7% (group A: non-diabetic), 226 with HbA1c ≥ 5.7% and <6.5% (group B: prediabetic) and 333 with HbA1c ≥ 6.5% (group C: diabetic). Biochemical and hematological parameters were compared among the three groups. An analysis of variance was performed to determine the correlations of the parameters among the groups. The ALB and sodium (Na) levels were significantly lower in group C than in groups A (ALB: 3.8 g/dL vs. 4.1 g/dL, *p* < 0.0001, Na: 138.4 mmol/L vs. 139.3 mmol/L, *p* < 0.001) and B (ALB: 3.8 g/dL vs. 4.0 g/dL, *p* < 0.0001, Na: 138.4 mmol/L vs. 139.6 mmol/L, *p* < 0.0001), whereas the RDW-standard deviation (RDW-SD) and urea were increased in group C as compared to group A (RDW: 45.8 vs. 43.9 fL, *p* < 0.0001, urea: 55.6 mg/dL vs. 38.5 mg/dL, *p* < 0.0001). The mean platelet volume (MPV) was increased in group C as compared to group A (9.3 fL vs. 9.1 fL, *p* < 0.05, respectively). Τhe increase in RDW-SD in group A as compared to B and C demonstrates the impact of hyperglycemia on red blood cells. Albumin and RDW might improve risk assessment for the development of diabetes. These results highlight the potential role of these parameters as an indication for prediabetes that would alert for measurement of HbA1c.

## 1. Introduction

Diabetes mellitus, a chronic metabolic disorder characterized by persistent hyperglycemia and affecting millions of people worldwide, results either from the body’s inability to produce enough insulin (Type 1 diabetes) or from the body’s cells’ ineffective use of insulin (Type 2 diabetes) [1]. The prevalence of diabetes is rising, affecting over 10.5% of the global adult population [2]. Early diagnosis and treatment are crucial for preventing the severe complications associated with this disease, such as cardiovascular diseases, neuropathy, retinopathy and nephropathy [3]. The diagnostic approach to diabetes typically includes several methods: fasting plasma glucose (GLU) tests, oral glucose tolerance testing and the measurement of glycated hemoglobin (HbA1c), which provides an average blood glucose level over the past two to three months [4,5,6]. This multi-faceted diagnostic approach is essential for the early detection and effective management of diabetes.

The HbA1c test is particularly valuable in the diagnosis and monitoring of diabetes because it provides a long-term picture of blood glucose homeostasis. It is not affected by short-term fluctuations in blood glucose levels caused by diet, stress, or illness. The American Diabetes Association states that an HbA1c level of 6.5% or higher on two separate tests confirms a diagnosis of diabetes, while levels between 5.7% and 6.4% indicate prediabetes, a state of increased risk for developing diabetes [1].

Red cell distribution width (RDW) is a hematological parameter that quantifies the variability in the size of circulating erythrocytes. Typically expressed as a percentage, RDW is calculated from the mean corpuscular volume (MCV) and is part of a standard complete blood count (CBC). Elevated RDW values indicate a greater degree of variability in the size of red blood cells, which can arise from various pathological conditions. In recent years, RDW has gained attention as a marker for several diseases beyond anemia. Specifically, its predictive and prognostic role in hepatic injury and chronic liver disease, as well as in Kawasaki disease, has been investigated [7,8]. Elevated RDW has been independently associated with increased mortality and adverse outcomes in patients with cardiovascular disease, chronic kidney disease, and sepsis [9,10]. The underlying mechanisms linking RDW to these conditions are not entirely understood, but it is hypothesized that RDW reflects systemic inflammation and oxidative stress, both of which are common in chronic diseases [11,12]. Inflammation can alter erythropoiesis and red blood cell survival, leading to increased heterogeneity in cell size. Similarly, oxidative stress can damage red blood cells, contributing to increased RDW [12].

In the context of diabetes, elevated RDW has been observed in patients with poor glycemic control and is associated with complications such as diabetic retinopathy, nephropathy and cardiovascular disease [13,14]. The exact relationship between RDW and diabetes is still under investigation, but it is believed that chronic hyperglycemia and associated metabolic disturbances contribute to the increased RDW seen in diabetic patients. Studies have suggested that RDW could serve as an early marker for prediabetes as well as diabetic complications, providing clinicians with a valuable tool for risk stratification and management [15,16].

Albumin (ALB) is a vital protein in human plasma, accounting for approximately 60% of the total plasma protein content. It is primarily produced by the liver and has several important physiological functions, including the maintenance of colloidal osmotic pressure, the binding and transport of various substances, and antioxidant properties [17,18]. The measurement of albumin levels in serum and urine is an important aspect of clinical diagnostics, particularly in the context of chronic diseases such as diabetes. Hypoalbuminemia on admission or the development of hypoalbuminemia during hospitalization has been associated with poor prognosis in hospitalized patients [19]. In relation to RDW, an elevated ratio of RDW to ALB has been found to be associated with an increased risk of peripheral artery disease in diabetic patients [20].

Moreover, serum albumin levels can also provide insights into the nutritional and inflammatory status of diabetic patients. Low serum albumin levels are associated with poor nutritional status, chronic inflammation, and increased risk of complications in diabetes [21,22]. Monitoring serum albumin levels, alongside urine albumin excretion, can therefore provide a more comprehensive assessment of a diabetic patient’s health status and help guide therapeutic interventions [23,24].

Given the significance of HbA1c, albumin and RDW as biomarkers in diabetes, exploring the correlations between these parameters might provide deeper insights into the disease’s pathophysiology and its complications. HbA1c reflects long-term glycemic control and is a key diagnostic and monitoring tool for diabetes. Serum albumin levels provide additional information on nutritional and inflammatory status. Elevated RDW, on the other hand, is associated with systemic inflammation and oxidative stress, conditions commonly seen in diabetes and its complications.

Investigating the correlations between these biomarkers in diabetic and non-diabetic individuals can help identify patterns that may improve our understanding of the disease. For instance, higher HbA1c levels may be associated with increased RDW and hypoalbuminemia, reflecting poor glycemic control and its impact on red blood cell morphology and kidney function. Conversely, non-diabetic individuals may exhibit balanced RDW and stable albumin levels, indicating better overall health and less systemic inflammation.

Based on the above, this study aimed to investigate the correlation of HbA1c with ALB, urea, sodium (Na), MCV, RDW, RDW-SD, platelets (PLT), mean platelet volume (MPV) and platelet distribution width (PDW) in diabetic, prediabetic and non-diabetic patients.

## 2. Results

Table 1 shows the mean values of hematologic and biochemical parameters in the groups.

This study found no differences between the mean values of PLT and MCV in group A, group B and group C, respectively. The *p*-value for each parameter was greater than 0.05, indicating no significant difference.

There were differences among the mean values of urea in group A, group B and group C, respectively.

There was a difference between the mean values of RDW-SD in group A and group B, respectively. Significant differences were found among the mean values of ALB Na, MPV, RDW and RDW-SD in group A and group C, respectively. There were also differences among the mean values of ALB, Na and PDW in group B and group C, respectively.

ANOVA was performed to test whether the outcomes of two or more groups differed significantly from each other. (Table 2 and Table 3).

In Table 2, a one-way ANOVA revealed that there was a statistically significant difference in the mean HbA1c score between at least two groups. A difference in the mean RDW–SD scores among the study groups was also revealed. The mean scores of MCV, RDW, PLT, MPV and PDW were not significantly different among the three groups.

Table 3 presents the one–way ANOVA analysis of the biochemical parameters among the three study groups. There was a difference in the mean ALB scores among the groups. There was also a difference in the mean urea scores among the study groups. There was a difference in the mean Na scores among the groups.

Pearson’s correlation coefficient (PCC) was calculated to assess the linear correlation of HbA1c and the ages of the participants in all the groups with the hematological and biochemical parameters (Table 4).

Table 4 shows a correlation of HbA1c with ALB, Na and urea. Age correlates significantly with ALB, urea, RDW, RDW-SD, MPV and PDW. The linear correlations are characterized as mild, because they show low PCC values.

## 3. Discussion

The present study aimed to investigate the relationship between the HbA1c levels and various hematological and biochemical parameters in a large cohort of participants. By categorizing the participants into three groups based on their HbA1c levels, we were able to identify several significant differences in key parameters, shedding light on how glycemic control might influence various aspects of health. Notably, this study is one of the first to comprehensively analyze these associations in such a large and diverse population, providing new insights into the intricate links between chronic glycemic levels and routine health markers.

### 3.1. Hematological Parameters

One of the primary findings of our study is that the mean values of platelet count (PLT) and the mean corpuscular volume (MCV) were not significantly different among the three groups, suggesting that HbA1c levels might not have a direct impact on these hematological parameters, which is in agreement with a recent study [25]. The consistency of PLT and MCV across groups A, B and C reinforces the notion that these particular measures are stable regardless of glycemic status. This stability may be particularly relevant in clinical practice, where these parameters are often used to assess general health and diagnose various conditions.

However, our analysis revealed significant differences in the red cell distribution width—standard deviation (RDW-SD) between groups A and B and groups A and C. Specifically, RDW-SD was higher in groups B and C as compared to group A. These findings suggest that higher HbA1c levels might be associated with greater variability in red cell size, which might reflect underlying erythropoietic stress [26]. RDW values have been shown to be elevated in several inflammatory conditions such as inflammatory bowel disease, systemic lupus erythematosus rheumatoid arthritis and psoriatic arthritis [27,28,29,30]. The underlying inflammation, which is known to play an important role in diabetic patients, could therefore affect RDW values [31].

In addition, the mean platelet volume (MPV) and platelet distribution width (PDW) were found to be significantly different when comparing groups A and C and groups B and C, respectively. MPV was higher in group C as compared to group A, and PDW was higher in group C as compared to group B. These findings indicate that platelet activation and size variability are potentially more pronounced in individuals with higher HbA1c levels, which is consistent with previous findings [32]. The increased reactivity of platelets in diabetic patients, however, is attributed to multiple factors, including hyperglycemia, hyperlipidemia, resistance to insulin and a more pronounced inflammatory and oxidative status along with increased expression of glycoprotein receptors and growth factors [33,34,35,36,37].

### 3.2. Biochemical Parameters

This study identified several significant differences in biochemical parameters among the groups. Urea levels increased significantly in the higher HbA1c groups. Elevated urea levels in higher HbA1c groups may indicate early kidney dysfunction, which is a common complication of diabetes. Another study suggested the use of urea nitrogen levels as a predictor of diabetes mellitus [38]. In this study, a concentration of urea nitrogen greater than 25 mg/dL was directly associated with a higher incidence of diabetes mellitus. Higher levels of urea nitrogen in people with chronic kidney disease have been shown to induce insulin resistance by activating E3 ubiquitin ligases that specifically conjugate ubiquitin to IRS-1, marking it for degradation by the ubiquitin-proteasome system [39]. Higher levels of urea nitrogen have also been shown to be associated with complications such as retinopathy in patients with type 2 diabetes [40].

ALB levels also showed significant differences among the groups. ALB levels were lower in group C than in groups A and B. This decrease in ALB with increasing HbA1c probably reflects the effect of chronic hyperglycemia on albumin permeability, as well as the chronic inflammation seen in diabetic patients [41]. The lower serum albumin levels can be partially attributed to the increased urinary albumin excretion due to hyperglycemia in type 2 diabetes patients [23].

Similarly, Na levels were lower in group C as compared to both groups A and B. Lower sodium levels in the higher HbA1c group could indicate a relative electrolyte imbalance, possibly due to altered renal function or shifts in fluid balance often seen in diabetes. In a study conducted among hypertensive individuals, lower Na levels were shown to be associated with an increased risk of developing diabetes [42].

### 3.3. Implications for Clinical Practice

The findings of this study have several implications for clinical practice. First, the lack of significant differences in PLT and MCV among HbA1c groups suggests that these hematological parameters are not influenced by glycemic homeostasis and can be reliably used in clinical assessments without adjustment for HbA1c levels. However, the significant differences in RDW-SD, MPV and PDW highlight the need for the careful monitoring of hematological changes in patients with poor glycemic control, as these parameters might signal an increased risk of complications such as anemia and thrombosis. Morphological changes in red blood cells are being extensively reviewed for their potential role as biomarkers of various disease states [43]. Therefore, the use of RDW, while different between diabetic patients and healthy adults, might be altered by various other conditions, and the clinical implications of this finding are limited [44].

The biochemical parameters that showed significant differences, particularly urea, ALB and Na, underline the importance of comprehensive metabolic monitoring in patients with varying degrees of glycemic control. The increase in urea levels in the higher HbA1c groups reinforces the need for strict glucose monitoring and management to prevent complications such as nephropathy. Additionally, the decrease in ALB and Na levels in the higher HbA1c groups suggests that routine liver and electrolyte monitoring could be beneficial in the management of patients with poor glycemic control to prevent and manage potential complications at an early stage.

Furthermore, based on our study, decreased Na and/or decreased ALB, and/or increased urea and/or increased RDW-SD in routinely tested patients should alert the clinician to request the measurement of HbA1c, as these findings may be associated with prediabetes or diabetes.

### 3.4. Limitations and Future Research

Despite these insightful findings, this study has limitations that should be acknowledged. The cross-sectional design limits the ability to establish causal relationships between HbA1c levels and the observed differences in hematological and biochemical parameters. Longitudinal studies are needed to determine the temporal relationship and causality, as well as the prognostic value of the measured parameters in terms of diabetes complications and survival time. In addition, although this study included a large and diverse sample, it did not account for potential confounding factors such as other medication use, diet and comorbid conditions, which could influence the observed parameters. The difference in the mean age among the groups might have played a role in the correlation with ALB and urea, although the correlation of age and these parameters was found to be mild, with low PCC values. Finally, the observed changes in the biochemical and hematological values in all groups, although statistically significant, are small in magnitude, which limits their clinical use.

Future research should focus on longitudinal studies to explore the causal relationships between glycemic control and the observed hematological and biochemical changes. Investigating the underlying mechanisms driving these changes, particularly the increase in RDW-SD, MPV and PDW, as well as the decrease in ALB and Na, will provide deeper insights into the pathophysiology of diabetes and its complications. Furthermore, studies examining the impact of specific interventions such as dietary changes, exercise, and medication adjustments on these parameters in individuals with varying HbA1c levels will help to develop targeted strategies to improve outcomes in diabetic patients.

## 4. Materials and Methods

### 4.1. Study Population

In this study, after obtaining written consent, 777 samples were collected from adult patients (372 women and 405 men, aged 19–85 years), who were examined and hospitalized at the General Hospital of Thessaloniki. The patients underwent a complete blood count, HbA1c and biochemical screening, including serum ALB, urea and sodium (Na). The samples were divided into three groups, based on their HbA1c levels: 218 participants with HbA1c < 5.7% (group A: non-diabetic, 116 women and 102 men, 19–82 years), 226 with HbA1c ≥ 5.7% and <6.5% (group B: prediabetic, 104 women and 122 men, 21–85 years) and 333 with HbA1c ≥ 6.5% (group C: diabetic, 152 women and 181 men, 20–85 years). Pearson’s chi-squared test was performed to assess the distribution of gender within the groups, revealing no statistically important variations (*p* = 0.154). All participants were not on diabetes-related medication at the time of the study. The participant characteristics are summarized in Table 5.

### 4.2. Hematologic Parameters

Fasted EDTA anticoagulated blood samples (2 mL) were collected from each participant in the morning hours and analyzed immediately. Samples with platelet clumps/clots were excluded.

A complete blood count was performed using the automated Beckman Coulter–DxH 800 Hematology Analyzer (Beckman Coulter, Miami, FL, USA). The presented parameters include MCV, RDW, RDW-SD, PLT, MPV and PDW. The count and the size of particles were determined using electrical impedance measurements according to the Coulter Principle. The mean corpuscular volume of the individual erythrocytes (MCV) was derived from the RBC histogram, multiplied by a calibration factor and expressed in femtoliters (fL). The size distribution range of the erythrocyte population (RDW and RDW-SD) was also derived from the RBC histogram. The RDW value is expressed as the coefficient of variation (%) and RDW-SD as a standard deviation in fL. The PLTs count was determined based on the Coulter Principle and expressed as 103 cells/μL. The mean platelet volume of MPV was derived from the PLT histogram, multiplied by a calibration factor and expressed in fL. The PLT size distribution spread (PDW) was derived from the PLT histogram and expressed as the coefficient of variation (%).

### 4.3. Biochemical Parameters

For the biochemical analysis, whole-blood samples (10 mL) were collected from each participant in the morning hours after overnight fasting. The samples were allowed to clot at room temperature for 20 min and were centrifuged at 3000 rpm for a total of 10 min. The separated serum samples were analyzed immediately and measured at least twice. Hemolyzed samples were excluded.

The ALB, urea and Na concentrations were measured using the Abbot Architect c16000 Analyzer (Abbott, Abbott Park, Chicago, IL, USA). The ALB and urea were determined by colorimetric methods. ALB levels were expressed in gr/dL and urea levels were expressed in mg/dL. Na was determined using an integrated chip technology (ICT) module based on a potentiometric method (ISE-Ion selective electrodes) and expressed in mmol/L.

EDTA anticoagulated blood samples (2 mL) from each participant were also collected at the same time point and immediately analyzed for the measurement of HbA1c. Hemolyzed samples and samples with platelet clumps were excluded. The percentage of glycated hemoglobin was measured based on HPLC technology, using the Tosoh Automated Glycohemoglobin HLC-723G8 Analyzer (Tosoh Europe B.V., Rembrandt Toren, Amsterdam, The Netherlands).

### 4.4. Statistical Analyses

For the statistical analyses, a statistical software package (IBM Corp., released 2021: IBM Statistical Package for Social Sciences—SPSS for Windows, Version 28.0, Armonk, NY, USA) was used to calculate the mean, median, standard deviation (SD) and all correlations of glycated hemoglobin with hematologic and biochemical markers. ANOVA (Analysis of Variance) was used to examine the relationships between the measured parameters. Pearson’s correlation coefficient was calculated to assess the linear correlation of the hematologic and biochemical markers with HbA1c and the ages of the participants. Pearson’s chi-squared test was performed to determine the distribution of genders among the three groups. The significance level (*p*-value) was set at 0.05 for all analyses.

## 5. Conclusions

This study provides a comprehensive analysis of the relationship between HbA1c levels and various hematological and biochemical parameters. The significant differences in parameters such as RDW-SD, MPV, PDW, GLU, urea, ALB and Na among different HbA1c groups highlight the complex interplay between glycemic homeostasis and overall health. The progressive increase in RDW-SD from the normal glucose homeostasis group to the prediabetes and diabetes groups demonstrates the significant impact of hyperglycemia on red blood cells. These findings underscore the importance of holistic monitoring and management of patients with diabetes, emphasizing the need for regular and comprehensive assessment beyond just glucose levels to prevent and manage the wide range of complications associated with diabetes. 

## Figures and Tables

**Table 1:** Mean values of hematologic and biochemical parameters in the groups.

		Mean Values			*p*-Values		
Hematologic Parameters	Reference Range	Group A (±SD)	Group B (±SD)	Group C (±SD)	Group A with Group B	Group A with Group C	Group B with Group C
HbA1c	<5.7%	5.3 (±0.27)	6.0 (±0.22)	8.0 (±1.78)	<0.0001	<0.0001	<0.0001
MCV	80.0–95.0 fL	87.7 (±7.85)	88.1 (±7.04)	87.7 (±6.79)	0.2634	0.4741	0.2484
RDW	11.5–14.5%	14.6 (±3.00)	15.0 (±3.13)	15.2 (±2.86)	0.0916	0.0201	0.2964
RDW-SD	40.0–55.0 fL	43.9 (±5.32)	45.8 (±6.08)	45.8 (±6.48)	<0.0005	<0.0001	0.4480
PLT	150−400 10^3^/μL	241.1 (±73.37)	241.5 (±87.44)	242.5 (±89.98)	0.4809	0.4315	0.4547
MPV	9.0−13.0 fL	9.1 (±1.05)	9.2 (±1.16)	9.3 (±1.12)	0.1628	<0.05	0.2043
PDW	9.0−17.0 fL	16.9 (±0.73)	16.8 (±0.74)	16.9 (0.72)	0.1709	0.2701	<0.05
Biochemical parameters							
ALB	3.5–5.0 g/dL	4.1 (±0.73)	4.0 (±0.74)	3.8 (±0.72)	0.2491	<0.0001	<0.0001
Urea	15.0–50.0 mg/dL	38.5 (±23.12)	46.3 (±27.96)	55.6 (±36.15)	0.0008	<0.0001	<0.0005
Na	136.0–146.0 mmol/L	139.3 (±2.92)	139.6 (±3.56)	138.4 (±3.55)	0.1650	0.001	<0.0001

Abbreviations: ALB: albumin; MCV: mean corpuscular volume; MPV: mean platelet volume; Na: sodium; PDW: platelet distribution width; PLT: platelets; RDW: red cell distribution width; and RDW-SD: red cell distribution width—standard deviation.

**Table 2:** Analysis of the variance between the hematologic parameters and study groups.

Hematologic Parameters	Analysis of Variance					
	Source of Variation	Sum of Squares	Degrees of Freedom	Mean Square	F	*p*-Value
HbA1c	Between Groups	1091.72	2.00	545.86	393.25	0.000000001
	Within Groups	1074.37	774.00	1.39		
	Total	2166.09	776.00			
MCV	Between Groups	28.91	2.00	14.46	0.28	0.755147002
	Within Groups	39,825.57	774.00	51.45		
	Total	39,854.48	776.00			
RDW	Between Groups	37.22	2.00	18.61	2.10	0.123107809
	Within Groups	6858.61	774.00	8.86		
	Total	6895.84	776.00			
RDW-SD	Between Groups	551.31	2.00	275.65	7.52	0.000586034
	Within Groups	27,912.28	761.00	36.68		
	Total	28,463.58	763.00			
PLT	Between Groups	212.98	2.00	106.49	0.01	0.985168391
	Within Groups	4,575,244.06	642.00	7126.55		
	Total	4,575,457.04	644.00			
MPV	Between Groups	4.50	2.00	2.25	1.81	0.163926007
	Within Groups	960.34	774.00	1.24		
	Total	964.84	776.00			
PDW	Between Groups	1.50	2.00	0.75	1.42	0.242158585
	Within Groups	339.39	642.00	0.53		
	Total	340.89	644.00			

Abbreviations: HbA1c: glycated hemoglobin; MCV: mean corpuscular volume; MPV: mean platelet volume; PDW: platelet distribution width; PLT: platelets; RDW: red cell distribution width; and RDW-SD: red cell distribution width—standard deviation.

**Table 3:** Analysis of the variance between the biochemical parameters and study groups.

Biochemical Parameters	Analysis of Variance					
	Source of Variation	Sum of Squares	Degrees of Freedom	Mean Square	F	*p*-Value
ALB	Between Groups	10.78	2.00	5.39	16.24	0.0000001230
	Within Groups	256.44	773.00	0.33		
	Total	267.22	775.00			
Urea	Between Groups	38,545.47	2.00	19,272.74	20.61	0.0000000019
	Within Groups	702,132.53	751.00	934.93		
	Total	740,678.00	753.00			
Na	Between Groups	189.65	2.00	94.82	8.72	0.0001819390
	Within Groups	7844.30	721.00	10.88		
	Total	8033.95	723.00			

Abbreviations: ALB: albumin; Na: sodium.

**Table 4:** Pearson’s correlation coefficient for HbA1c and age with hematologic and biochemical markers.

Pearson’s Correlation Test										
		ALB	Na	Urea	MCV	RDW	RDW-SD	PLT	MPV	PDW
HbA1c	PCC	−0.23	−0.174	0.148	−0.049	0.152	0.016	−0.059	0.035	0.068
	*p*	<0.0001	<0.0001	<0.0001	0.169	<0.0001	0.61	0.132	0.334	0.084
Age	PCC	−0.173	0.015	0.278	0.076	0.023	0.198	−0.083	0.085	0.154
	*p*	<0.0001	0.727	<0.0001	0.058	0.515	<0.0001	0.062	0.036	0.001

**Table 5:** Participant characteristics.

Participant Characteristics	Group A n = 218 (%)	Group B n = 226 (%)	Group C n = 333 (%)	Total n = 777 (%)
**Gender**				
Male	102 (25.19)	122 (30.12)	181 (44.69)	405 (100.00)
Female	116 (31.18)	104 (27.96)	152 (40.86)	372 (100.00)
	**mean (±SD)**			
**Age**	54 (±14.23)	63 (±13.56)	66 (±13.83)	62 (±14.63)

Abbreviations: SD, standard deviation.

## Data Availability

The data presented in this study are available on request from the corresponding author due to ethical restrictions.
